# Pandemic Preparedness: Maintaining Adequate Immune Fitness by Attaining a Normal, Healthy Body Weight

**DOI:** 10.3390/jcm11143933

**Published:** 2022-07-06

**Authors:** Pantea Kiani, Kiki E. W. Mulder, Jessica Balikji, Aletta D. Kraneveld, Johan Garssen, Joris C. Verster

**Affiliations:** 1 Division of Pharmacology, Utrecht Institute for Pharmaceutical Sciences, Faculty of Science, Utrecht University, 3584 CG Utrecht, The Netherlands; p.kiani@uu.nl (P.K.); k.e.w.mulder@students.uu.nl (K.E.W.M.); j.balikji@uu.nl (J.B.); a.d.kraneveld@uu.nl (A.D.K.); j.garssen@uu.nl (J.G.); 2 Global Centre of Excellence Immunology, Nutricia Danone Research, 3584 CT Utrecht, The Netherlands; 3 Centre for Human Psychopharmacology, Swinburne University, Melbourne, VIC 3122, Australia

**Keywords:** pandemic preparedness, SARS-CoV-2, COVID-19, immune fitness, bodyweight, body mass index, age, height, sex

## Abstract

In addition to developing effective medicines and vaccines, pandemic preparedness also comprises general health-related, behavioral, and psychological aspects related to being more resistant in the case of future pandemics. In the context of the 2019 coronavirus (COVID-19) pandemic, recent research revealed that reduced perceived immune fitness was the best predictor of reporting more frequent and more severe COVID-19 symptoms. Up until now (June 2022), during the COVID-19 pandemic, the majority of patients who have been hospitalized were characterized as being overweight. It is therefore essential to further evaluate the relationship between body mass index (BMI) and immune fitness. This was performed by analyzing pooled data from previously published studies, conducted among N = 8586 Dutch adults. It was hypothesized that attaining a normal, healthy body weight is associated with optimal perceived immune fitness. The analysis revealed that a deviation from normal weight (i.e., having a BMI outside the range of 18.5 to 24.9 kg/m^2^) was associated with significantly reduced perceived immune fitness, as assessed with the immune status questionnaire and a single item perceived immune fitness scale. The effects were significant for both underweight and overweight groups and most pronounced for the obese groups. The results suggest that attaining a normal, healthy body weight might significantly contribute to maintaining adequate perceived immune fitness. Therefore, attaining a normal body weight might be an essential component of pandemic preparedness and should be supported by creating awareness and promoting the importance of regular exercise and the consumption of healthy food.

## 1. Introduction

Preparedness of the population is of great importance to being more resistant to potential future pandemics. Besides developing effective medicines and vaccines, pandemic preparedness also comprises general health-related, behavioral, and psychological aspects such as coping with stress [1,2]. A resilient immune fitness (i.e., an adequate immune system) refers to the capacity to adapt to external health challenges by establishing an appropriate immune response to prevent or resolve diseases. Poor immune fitness is caused partly by genetic predisposition and personal characteristics such as age, sex, and underlying conditions, but it is also significantly impacted by lifestyle factors such as daily diet, (lack of) physical exercise, chronic stress, and poor sleep [3,4,5]. Recently, Kiani et al. [6] evaluated the impact of reduced perceived immune fitness on the presence and severity of 2019 coronavirus (COVID-19) complaints. The stepwise regression analysis by Kiani et al. [6], including most of these factors, revealed that perceived immune fitness before the pandemic was the most important predictor of the number and severity of COVID-19 symptoms. Maintaining a resilient immune fitness seems to be essential to being prepared for future pandemics.

The most prominent characteristics of patients hospitalized with COVID-19, in both the regular care unit and the intensive care unit, are older age and having underlying diseases [7,8]. In particular, obesity is a frequently mentioned risk factor for testing positive for SARS-CoV2 [9,10], experiencing more severe COVID-19 symptoms [11,12,13,14,15], increased rates of hospitalization [11,12,13,14,15,16,17], and significantly higher mortality rates [18,19]. Taken together, obesity is an important factor impacting the disease course of COVID-19, and as such, its relationship with immune fitness warrants further investigation.

Obesity is an increasing worldwide health concern, which affects over 650 million adults (circa 13% of the world’s adult population) and 124 million children and adolescents worldwide [20]. There are different factors that can contribute to weight gain or weight loss. For example, some medications for psychiatric conditions, genetic syndromes, and hypothalamic and endocrine diseases can influence body weight, which cause low-grade and chronic inflammatory processes in the human body [21]. In addition, lifestyle factors (e.g., exercise and physical activity) and nutrition impact body weight [22,23,24]. An established method to determine body weight status is to calculate the body mass index (BMI, in kg/m^2^), considering height and weight [25,26]. The World Health Organization (WHO) definition of overweight (pre-obesity) is a BMI ≥ 25 kg/m^2^ but below 30 kg/m^2^, and that for obesity is a BMI ≥ 30 kg/m^2^ [27]. The potential impact of obesity on the number of patients infected by COVID-19 that require hospitalization may be particularly relevant to the United States [28]. This is because the nationwide prevalence of obesity in the US (about 40%) is much higher compared with most other parts of the world, including China (6.2%) and Europe (20–25%) [29].

The increased risk for infection and enhanced symptom severity among patients who are obese is related to low-grade chronic inflammation [30,31]. Obesity leads to mechanical and inflammatory adverse pulmonary effects, and patients who are obese are more likely to suffer from respiratory failure [32]. Other studies suggested that increased visceral fat accumulation (i.e., abdominal adiposity) is related to worse clinical outcomes among patients with COVID-19 [33,34].

While several studies have previously reported on the association between biomarkers of the immune system and BMI [35,36,37], this is the first study that evaluated the relationship between body weight and self-reported, perceived immune fitness. The latter is important as previous research revealed that perceived immune fitness is the strongest predictor for the presence and severity of COVID-19 symptoms [6]. It was hypothesized that a BMI that deviates from the normal weight group (i.e., 18.5 to 24.9 kg/m^2^) is associated with poorer perceived immune fitness.

## 2. Methods

For the present analysis, data on sex, height, age, and perceived immune fitness from various studies were pooled [38,39,40,41,42,43,44,45,46,47,48,49]. Studies were included if immune fitness was assessed with the immune status questionnaire (ISQ) and/or single item perceived immune fitness scale. All subjects provided informed consent to participate in the original studies. No ethics approval was needed for this pooled data analysis.

Past 12-month immune fitness was measured with the Immune Status Questionnaire (ISQ) [50]. The ISQ comprises seven items including ‘common cold’, ‘diarrhea’, ‘sudden high fever’, ‘headache’, ‘muscle and joint pain’, ‘skin problems (e.g., acne and eczema)’, and ‘coughing’. Subjects reported how frequently they experienced these immune-related complaints by choosing between the answering possibilities ‘never’, ‘sometimes’, ‘regularly’, ‘often’, and ‘(almost) always’. The overall ISQ score, after recoding, ranges from 0 (poor) to 10 (excellent). Momentary perceived immune fitness was measured with a single-item scale ranging from 0 (poor) to 10 (excellent) [50].

Body mass index (BMI, kg/m^2^) was computed from self-reported weight and height data. According to the World Health Organization BMI classification [27], subjects were allocated to one of the following groups: (1) underweight group (BMI < 18.5), (2) normal-weight group (BMI 18.5–24.9), (3) overweight group (BMI 25.0–29.9), (4) obesity class I group (BMI 30.0–34.9), (5) obesity class II group (BMI 35.0–39.9), or the (6) obesity class III group (BMI ≥ 40).

The data were analyzed with SPSS (IBM Corp. Released 2013. IBM SPSS Statistics for Windows, Version 28.0. Armonk, NY, USA: IBM Corp.). The mean and standard deviation of ISQ and momentary perceived immune fitness were computed for each variable and each BMI group. Differences between BMI groups and the normal weight group were tested for statistical significance with the Independent Samples Kruskal–Wallis Test and considered significant if *p* < 0.01 (after Bonferroni’s correction for multiple comparisons). For each BMI group, percentages were calculated of the number of subjects with a score <6, indicating reduced immune fitness. The percentages of the BMI groups were compared with those of the normal weight group using the “N-1” Chi-squared test, as recommended by Campbell [51] and Richardson [52]. The calculations were conducted using the MedCalc Software Ltd. Comparison of proportions calculator, available at https://www.medcalc.org/calc/comparison_of_proportions.php (Version 20.106; accessed 23 April 2022). Differences from the normal weight group were considered significant if *p* < 0.01 (after Bonferroni’s correction for multiple comparisons). Differences between men and women were evaluated with the Independent Samples Mann–Whitney U test and considered significant if *p* < 0.05.

## 3. Results

**Figure 1:**
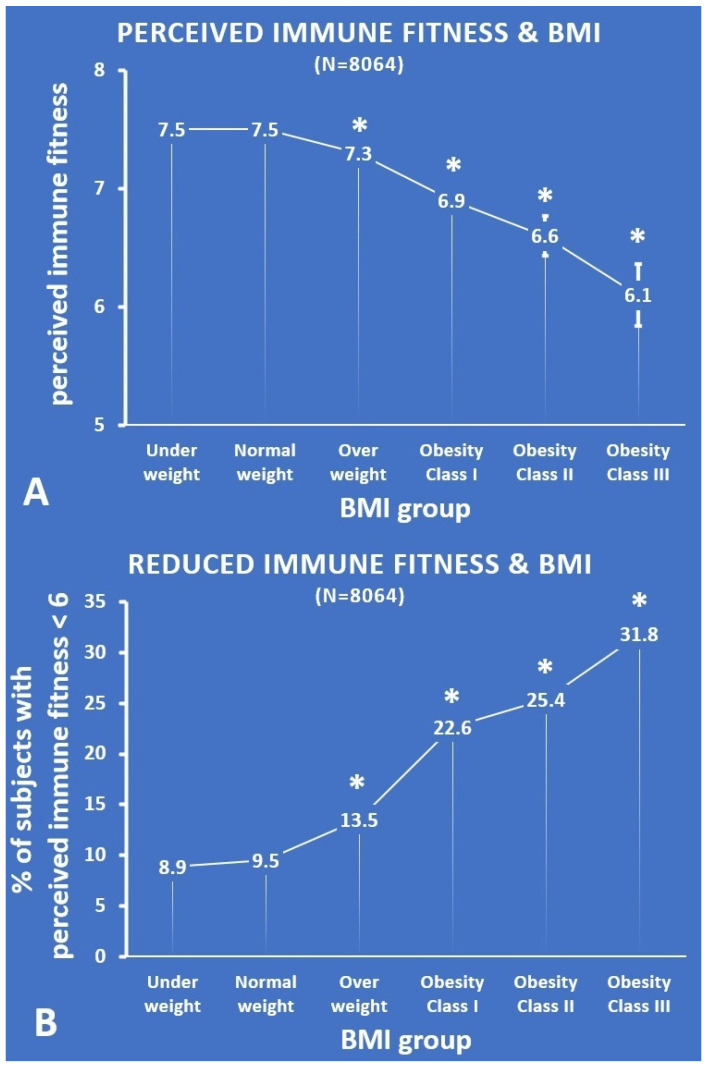
Momentary perceived immune fitness according to BMI group. (**A**) Each BMI group’s mean (SE) momentary perceived immune fitness score; (**B**) the percentage of subjects with a momentary perceived immune fitness score < 6. Significant differences (*p* < 0.01, after Bonferroni’s correction) compared with the normal weight group are indicated by *. Abbreviation: BMI = body mass index.

The data of N = 8586 subjects were analyzed. Their mean (SD) age was 32.4 (16.7), with an age range of 18 to 103 years old. The sample comprised 32.3% of men. Subjects reported a mean (SD) weight of 72.5 (15.5) kg and a mean (SD) height of 1.74 (0.09) m. A total of N = 8064 subjects completed the momentary perceived immune fitness scale, and N = 4263 subjects completed the ISQ. Their mean (SD) momentary perceived immune fitness score was 7.4 (1.7) and mean (SD) ISQ of 6.9 (2.5), both with a range from 0 to 10. Table 1 and Figure 1 summarize the data on perceived immune fitness.

Compared with the normal weight group, momentary perceived immune fitness was significantly lower for the overweight group (*p* < 0.001, effect size (ES) = 0.31) and the obesity class I (*p* < 0.001, ES = 0.20), class II (*p* < 0.001, ES = 0.10), and class III groups (*p* < 0.001, ES = 0.04). With increasing BMI, the perceived immune scores become lower (see Figure 1A). Subjects who were underweight did not rate their momentary perceived immune fitness as significantly lower than the normal weight group. The percentages of subjects that reported reduced momentary perceived immune fitness (i.e., a score below 6), i.e., those with reduced immune fitness, were summarized according to BMI group and are shown in Figure 1B. The percentage of subjects that reported reduced momentary perceived immune fitness was significantly greater for the overweight group and the obesity class I, II, and III groups (*p* < 0.001 for all paired comparisons). With increasing BMI, the percentage of subjects who reported reduced momentary perceived immune scores steadily increased (see Figure 1B). Again, the underweight group did not significantly differ from the normal weight group.

**Figure 2:**
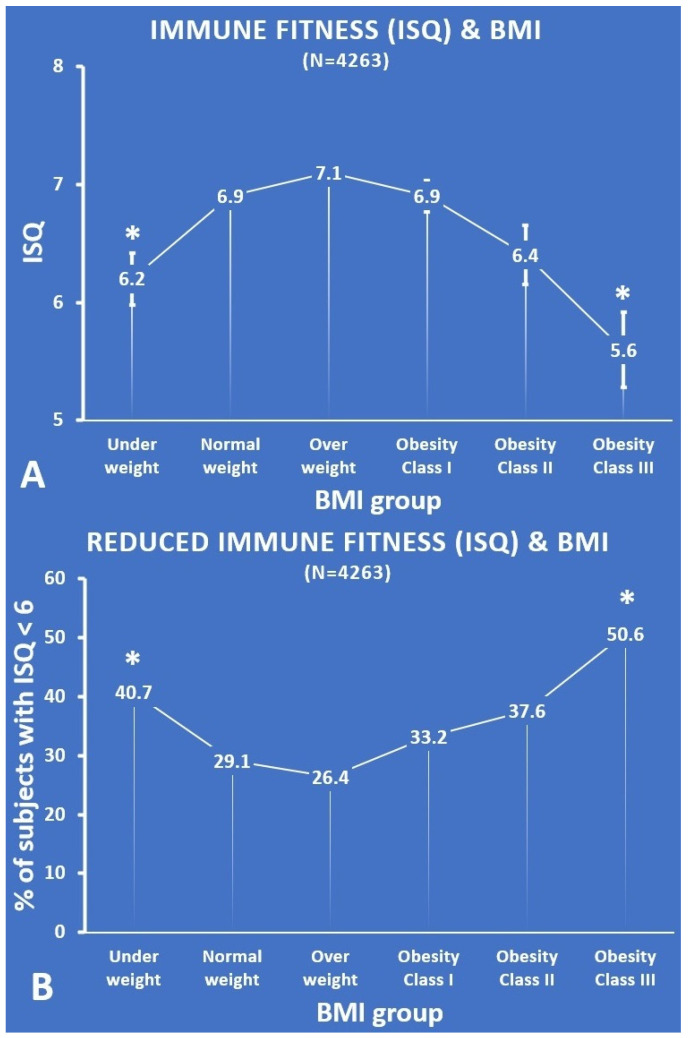
ISQ according to BMI group. (**A**) Each BMI group’s mean (SE) ISQ score; (**B**) the percentage of subjects with an ISQ score < 6. Significant differences (*p* < 0.01, after Bonferroni’s correction) compared with the normal weight group are indicated by *. Abbreviations: ISQ = Immune status Questionnaire, BMI = body mass index.

Table 2 and Figure 2 summarize the data on past year’s perceived immune fitness as assessed with the ISQ.

Compared with the normal weight group, the ISQ was significantly lower for the underweight group (*p* = 0.001, ES = 0.15) and the obesity class III group (*p* < 0.001, ES = 0.25). Thus, an inverse U-curve was found for the relationship between ISQ and BMI (see Figure 2A).

The percentages of subjects that had reduced immune fitness according to the ISQ (i.e., a score below 6) were significantly greater for the underweight group (*p* = 0.005) and the obesity class III group (*p* = 0.001). The difference between the normal weight group and the overweight group (*p* = 0.090), the obesity class I group (*p* = 0.087), and the obesity class II group (*p* = 0.034) did not reach statistical significance. With increasing BMI, the percentage of subjects who reported reduced immune scores on the ISQ followed a U-shape (see Figure 2B).

Finally, men and women differed significantly in BMI and immune fitness. The mean ±SD BMI of women was significantly lower than the BMI of men (23.6 ± 4.6 vs. 24.6 ± 4.3, respectively, *p* < 0.001, ES = 0.16). Overall, women rated their momentary perceived immune fitness significantly lower than men (7.2 ± 1.7 vs. 7.8 ± 1.5, for women vs. men, respectively, *p* < 0.001, ES = 0.16). In line with this, the ISQ scores of women were significantly lower than those of men (6.4 ± 2.6 vs. 7.7 ± 2.2, respectively, *p* < 0.001, ES = 0.25). The sample size of the obesity class II and obesity class III were too small to allow for a reliable evaluation of sex possible differences in momentary perceived immune fitness and ISQ scores between the BMI groups and the normal weight group.

## 4. Discussion

This is the first study relating perceived immune fitness to BMI. The analysis revealed that deviation from a normal weight (i.e., having a BMI outside the range of 18.5 to 24.9 kg/m^2^) was associated with significantly reduced perceived immune fitness, as assessed with either the ISQ or the momentary perceived immune fitness scale. The momentary perceived immune fitness effects were significant for both underweight and overweight groups and most pronounced for the obesity groups. The results suggest that attaining a normal body weight can significantly contribute to maintaining adequate immune fitness and should therefore be supported.

Individuals can influence lifestyle factors to improve their immune fitness and thus their pandemic preparedness. For example, research has shown a significant correlation between alcohol consumption and perceived immune fitness [53,54,55]. In the same subjects, a significant correlation was also observed between perceived immune fitness and the presence and severity of COVID-19 symptoms [53,54,55]. Reducing alcohol consumption can thus help to improve the perceived immune fitness. Another notable factor that impacts BMI is physical activity. Exercise and physical activity support the immune system to stay balanced and resilient, i.e., maintaining adequate immune fitness [22,56], and has been demonstrated in relation to several diseases, such as obesity, diabetes, and cardiovascular disease [56].

Alternatively, reducing stress may reduce or prevent low-grade inflammation [57,58] and may avoid the negative impact of stress on other affected lifestyle factors, such as unhealthy eating habits and poor sleep quality [59]. Additionally, diet can significantly prevent or induce low-grade inflammation [60,61]. Eating a high amount of ultra-processed energy-dense and other unhealthy food with a low amount of healthy nutrients can cause low-grade inflammation and increase the risk of infections and other immune-related disorders [62,63]. In this context, it has been shown that a lifestyle switch to no exercise and consuming an unhealthy diet for only two weeks has already resulted in reduced insulin sensitivity, a higher level of body fat, and low-grade inflammation [64]. Alternatively, switching to a Mediterranean diet already showed positive health effects after only one week [65,66]. Taken together, regular exercise and a healthy diet can prevent low-grade inflammation [67] and can help attain normal, healthy body weight, thereby improving immune fitness.

As described above, there are several factors that can influence bodyweight. In addition, it is important to note that only attaining a normal, healthy bodyweight will likely not be sufficient to promote adequate immune fitness. Mental health (e.g., stress reduction), healthy nutrition, and adequate and regular physical activity are factors that can directly improve immune fitness [57,58,59,60,61,62,63]. Therefore, in terms of pandemic preparedness, attaining a normal, healthy BMI should be viewed as one of several measures that could be adopted by individuals.

This study has a couple of limitations. First, the data were self-reported and, in part, retrospective, which may have introduced bias (e.g., recall bias). Second, no information was considered regarding possible reasons for differences in BMI or reported immune fitness (e.g., diet status). As the dataset comprised a combination of different studies with different aims, such data were not collected consistently across the studies. Third, the male groups for underweight and obesity classes II and III were too small to allow for a meaningful comparison between men and women. Such a comparison should be the aim of future research when larger samples are collected. Finally, besides BMI, there are several other correlates of perceived immune fitness (e.g., underlying disease). These were not presented here, as these were beyond the scope of the current article, and the data on these participant characteristics were not (consistently) collected across studies.

## 5. Conclusions

The preparedness for future pandemics should not solely rely on developing vaccines and medicines. Previous research revealed that adequate perceived immune fitness is the best predictor of experiencing less and less severe COVID-19 symptoms when infected with SARS-CoV-2. The current findings demonstrate that attaining a normal body weight could help in attaining optimal perceived immune fitness.

## Figures and Tables

**Table 1:** Momentary perceived immune fitness according to BMI group.

BMI Group	Under Weight	Normal Weight	Over Weight	Obesity Class I	Obesity Class II	Obesity Class III
N	361	5398	1622	475	142	66
Mean (SD)	7.5 (1.5)	7.5 (1.5)	7.3 (1.7) *	6.9 (2.2) *	6.6 (2.0) *	6.1 (2.1) *
% < 6	8.9	9.5	13.5 *	22.6 *	25.4 *	31.8 *

Mean and standard deviation (SD) or percentages are shown. Differences from the normal weight group are considered statistically significant if *p* < 0.01 (after Bonferroni’s correction for multiple comparisons), indicated by *. % < 6 = subjects with a momentary perceived immune fitness score < 6, suggesting reduced immune fitness. Abbreviations: BMI = body mass index, SD = standard deviation.

**Table 2:** ISQ according to BMI group.

BMI Group	Under Weight	Normal Weight	Over Weight	Obesity Class I	Obesity Class II	Obesity Class III
N	130	2259	1233	434	138	69
Mean (SD) ISQ	6.2 (2.5) *	6.9 (2.5)	7.1 (2.5) *	6.9 (2.7)	6.4 (2.9)	5.6 (2.7) *
% < 6	40.7 *	29.1	26.4	33.2	37.6	50.6 *

Mean and standard deviation (SD) or percentages are shown. Differences from the normal weight group are considered statistically significant if *p* < 0.01 (after Bonferroni’s correction for multiple comparisons), indicated by *. % < 6 = subjects with an ISQ score <6, suggesting reduced immune fitness. Abbreviations: BMI = body mass index, SD = standard deviation, ISQ = immune status questionnaire.

## Data Availability

The data are available from the corresponding author upon reasonable request.
